# Intracystic papillary carcinoma in a male as a rare presentation of breast cancer: a case report and literature review

**DOI:** 10.1186/1752-1947-3-13

**Published:** 2009-01-13

**Authors:** Laszlo Romics, M Emmet O'Brien, Norma Relihan, Fionnuala O'Connell, H Paul Redmond

**Affiliations:** 1 Department of Surgery, Cork University Hospital, University College Cork, Wilton Road, Cork, Ireland; 2 Faculty of Medicine and Health, University College Cork, Cork, Ireland; 3 Department of Pathology, Cork University Hospital, University College Cork, Wilton Road, Cork, Ireland

## Abstract

**Introduction:**

The term "intracystic papillary ductal carcinoma *in situ*" has recently changed and is now more appropriately referred to "intracystic papillary carcinoma". Intracystic papillary carcinoma in men is an extremely rare disease with only a few case presentations published in the literature so far.

**Case presentation:**

We discuss a case of a 44-year-old Caucasian man with an intracystic papillary carcinoma treated with simple mastectomy, sentinel lymph-node biopsy and contralateral risk-reducing mastectomy. These were followed by adjuvant radiotherapy of the breast.

**Conclusion:**

Triple assessment (i.e. clinical examination and radiological and histological assessment) with a high level of clinical suspicion is necessary to diagnose intracystic papillary carcinoma in men due to its rarity. Furthermore, genetic testing and risk-reducing mastectomy should also be considered in cases of a strong family history for male breast cancer.

## Introduction

Breast carcinoma in men is rare; it represents 0.6% of all breast carcinomas and less than 1% of all malignancies in men. Male breast cancer has an incidence of one per 100,000 per annum. Overall survival rates for men with breast carcinoma, stratified by stage of disease, are lower than for women with breast carcinoma. However, these differences are most likely due to the higher age distribution of male patients and the lower life expectancy of men in the general population [1].

Intracystic papillary carcinoma (IPC) is a rare form of breast cancer, accounting for 0.5–1% of all breast cancers. It typically occurs in older women and has an excellent prognosis. The reported 10-year survival rate for IPC is 100%, the recurrence-free survival rate is 96% and 77% at 2 and 10 years, respectively [2].

Here, we report the case of intracystic papillary ductal carcinoma *in situ *(DCIS)/carcinoma of the breast in a 44-year-old male patient.

## Case presentation

A 44-year-old Caucasian man presented to the breast clinic with a 3-week history of a swelling in his left breast. He also had a significant family history for breast cancer including a maternal grandmother, two of his maternal aunts and a maternal first cousin diagnosed with breast cancer. On examination, a well-circumscribed, 2.5 cm swelling was palpable within an area of gynecomastia on the left chest wall. Sonographically, a cystic mass with internal echoes was present without posterior acoustic shadowing. Aspiration of the lesion revealed uniformly blood-stained fluid and a residual swelling persisted. Cytology analysis of the aspirate confirmed the presence of atypical cells. Mammography showed a circumscribed mass in the sub-areolar region of the left breast with partially obscured margins. An irregular outline was noted on cranio-caudal view, but no spiculation or suspicious internal micro-calcifications were found. A core biopsy of the lesion revealed atypical ductal hyperplasia, but no evidence of malignancy was seen. In view of the atypical cells and residual swelling the lesion was excised.

**Figure 1:**
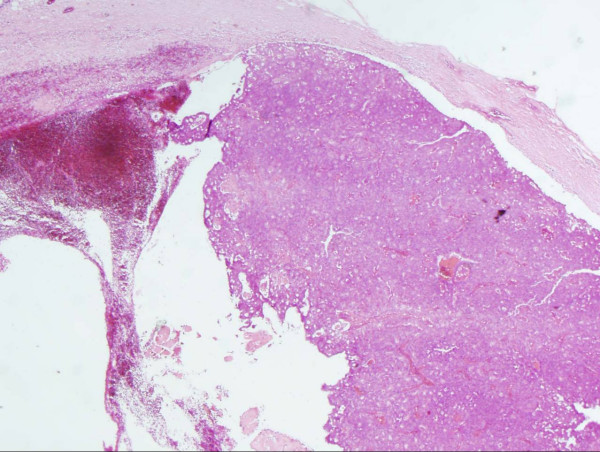
**Hematoxylin-eosin stain in excised tumours**. Low-power view illustrating a 2.5 × 1.8 × 1.2 cm lesion within a large cystic space surrounded by a thick fibrous capsule. Haemorrhage was also noted within the cyst with changes consistent with the prior biopsy.

**Figure 2:**
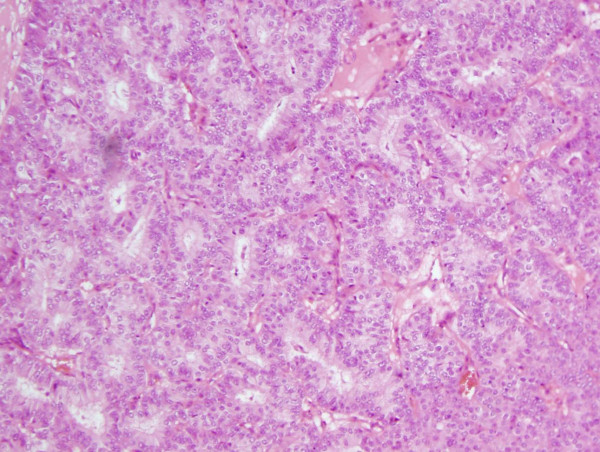
**Hematoxylin-eosin stain in excised tumours, At higher power, a papillary and solid proliferation of atypical cells of a uniform population were observed**. Fibrovascular cores were well visualized.

**Figure 3:**
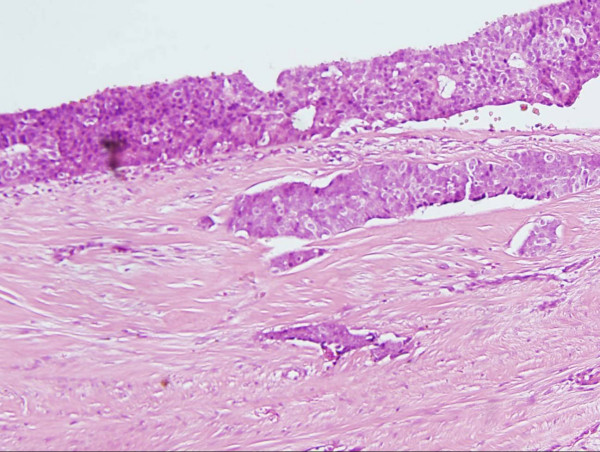
**Hematoxylin-eosin stain in excised tumours, At higher power, a papillary and solid proliferation of atypical cells of a uniform population were observed**. Fibrovascular cores were well visualized.

Histological analysis revealed a lesion 2.5 cm × 1.8 cm × 1.2 cm in size. The lesion comprised a papillary and solid proliferation of atypical cells within a large cystic space with a thick fibrous capsule (Figures 1, 2, 3) Haemorrhage was also noted within the cyst with changes consistent with the prior biopsy. The margins were clear; there was no evidence of stromal or fibrovascular invasion. The lesion displayed features of papillary DCIS and a diagnosis of intracystic papillary DCIS was made. Immunohistochemistry showed oestrogen receptor (ER) and progesterone receptor (PR) positivity.

Completion left mastectomy with sentinel lymph-node mapping was carried out. Contralateral risk-reducing mastectomy was also performed in view of the strong family history for breast cancer. No evidence of further disease was detected in the mammary tissue and the sentinel node was clear. Adjuvant radiotherapy (40 Gy in 25 fractions) was advised due to tumour extension close to deep margin.

Due to his significant family history for breast cancer, genetic testing was offered. Breast-cancer gene 2 (BRCA2) mutation had been identified in a maternal aunt recently on the exon 24 insertion called c9481_9482insA. It was very likely that this patient was a carrier of this BRCA2 mutation, which poses a small risk for him to develop prostate cancer. He was therefore advised to undergo prostate-specific antigen (PSA) blood testing on a yearly basis.

## Discussion

IPC is a rare malignancy of the breast; however, a relatively higher incidence range of 5–7.5% has been reported in men [3-5]. IPC in men is usually reported among those of an older age group (68 to 84 years) [4,6-9]; however, in our patient, IPC developed at a significantly younger age, which might be due to this patient likely carrying the BRCA2 mutation. The prognosis for this type of tumour is excellent [3,7]. In a study of 77 patients with IPC all patients were alive 10 years after their diagnosis and metastases occurred only in 4% of patients, but none of the patients with low-grade tumours were in this group [10].

The terminology applied to describe papillary breast lesions in the literature is relatively confusing. The traditional term "intracystic papillary carcinoma" generally refers to a localized lesion, *in situ *in a cystically dilated duct. Given the often marked stromal response surrounding these lesions, the distinction between *in situ *and invasive papillary carcinoma can be very difficult to make. Therefore, IPC had been divided it into three subgroups, which seems to correlate with the prognosis: IPC alone, IPC plus DCIS, and IPC with invasion [5]. In this manner, the term "papillary DCIS" would refer to a more diffuse process that involves multiple ducts as opposed to a localized lesion [5].

Recently, Hill et al., using myoepithelial cell staining, suggest a spectrum of progression from *in situ *disease to invasive disease, signifying that what appears to be DCIS on histology may potentially cause distant metastases [11]. The lack of an intact basal myoepithel cell layer can be identified by calponin, smooth-muscle myosin heavy chain (SMM-HC) cytoplasmic stains and by p63 nuclear stains. This "gold standard" method has a relatively high sensitivity and denotes the invasiveness of the tumour cells in malignant papillary breast lesions [11].

The diagnosis of IPC of the male breast should be made carefully. Triple assessment is essential and the goal is to achieve a preoperative diagnosis prior to surgery. The radiological diagnosis of IPC is relatively challenging. The typical sonographical appearance of IPC is a hypoechoic area with soft tissue echoes projecting from the wall of the cyst [6,7]. However, a relatively large amount of variation exists on ultrasounds from an intraductal (which might be associated with ductal dilatation) and a predominantly solid pattern with the intraductal or intracystic mass totally filling the duct [12]. Importantly, IPCs are highly vascular tumours demonstrating a characteristic flow pattern on colour-flow studies, which are sensitive to identifying even very small IPCs. A distinct vascular pedicle can be identified within the central core with branching vessels arborising within the mass [12].

The mammographic appearance of IPCs is less specific. Small IPCs are often mammographically negative, while larger lesions may resemble any other focal well-circumscribed dense mass on mammography [12]. Both can cause a minimal to moderate duct dilatation in a tapering band-like density pattern from the nipple towards the parenchyma. In addition, one report suggested the use of pneumocystography [8], and another MRI [9], in combination with mammography and ultrasound to diagnose IPC.

Fine-needle aspiration cytology and core biopsy are usually performed; however, the false negative results with cytology are relatively frequent [13]. Therefore, excisional biopsy should be carried out in all cystic lesions of the male breast which are suspicious on any of the above diagnostic modalities.

There are no clear guidelines about the management of IPC, which is due to various factors. On one hand, IPC is a rarity; on the other hand, the histopathological classification and detection of invasiveness in IPC is rather confusing [3,5]. In a recent review, Grabowski et al. [3] confirmed that surgery is the mainstay of treatment, which can be either conservation or mastectomy. Since the prognosis of IPC is excellent with low locoregional and distant recurrence rates, mastectomy is usually not necessary, unless it is technically unavoidable [3]. Axillary node metastasis can occur in up to 14% of the cases [3]; therefore, an axillary staging procedure or clearance is recommended by most authors [3,4,9]. Others argue that IPC should be generally regarded as an *in situ *disease; therefore axillary surgery is not recommended by these authors [6-8]. There has been no clear indication for adjuvant endocrine therapy, even among patients with oestrogen-receptor-positive tumours. The addition of hormonal treatment does not appear to have impacted the outcome [3]. On the contrary, Fayanju et al. recently reviewed the usual adjuvant treatment applied for IPC and found that patients with DCIS or microinvasive disease in association with IPC were more likely to receive radiotherapy and tamoxifen [14].

Between 4% and 40% of male breast cancers might result from autosomal dominant mutations, primarily BRCA1 or BRCA2 mutations [15]. Due to the high risk to our patient, contralateral risk-reducing mastectomy was also carried out. This reduces the incidence of contralateral breast cancer by approximately 95% but will not have an impact on overall survival of the patient [15].

## Conclusion

Triple assessment with a high level of clinical scepticismscepticism is necessary to diagnose IPC in a man, due to the rarity of the condition. The treatment of choice for this tumour is ample local excision. However, genetic testing and risk-reducing mastectomy should also be considered in cases of male breast cancer with a strong family history.

## Abbreviations

BRCA1 or BRCA2: breast-cancer gene 1 or 2; DCIS: ductal carcinoma *in situ*; ER: oestrogen receptor; IPC: intracystic papillary carcinoma; PR: progesterone receptor; PSA: prostate-specific antigen; SMM-HC: smooth-muscle myosin heavy chain

## Competing interests

The authors declare that they have no competing interests.

## Authors' contributions

LR reviewed the case notes and wrote up the manuscript. ME, OB and NR did the literature review and contributed to the completion of the manuscript. FOC carried out the histopathological analysis and HPR created the final version of the manuscript.

## Consent

Written informed consent was obtained from the patient for publication of this case report and accompanying images. A copy of the written consent is available for review by the Editor-in-Chief of this journal.
